# Clinical outcomes following switch from risankizumab to guselkumab in Crohn’s disease: a real-world case series

**DOI:** 10.1093/ecco-jcc/jjag089

**Published:** 2026-06-25

**Authors:** Amar Farkouh, Nabil Nathoo-Khedri, Alice Dufresne, Homa Danaie, Robert Battat

**Affiliations:** Centre Hospitalier de l‘Université de Montréal (CHUM), CHUM Research Center (CRCHUM), University of Montreal, Montreal, Canada; Centre Hospitalier de l‘Université de Montréal (CHUM), CHUM Research Center (CRCHUM), University of Montreal, Montreal, Canada; Faculty of Medicine, University of Montreal, Montreal, Canada; Faculty of Medicine, University of Montreal, Montreal, Canada; Centre Hospitalier de l‘Université de Montréal (CHUM), CHUM Research Center (CRCHUM), University of Montreal, Montreal, Canada; Division of Gastroenterology, Centre Hospitalier de l‘Université de Montréal (CHUM), Montreal, Canada

**Keywords:** Crohn’s disease, risankizumab, guselkumab, IL-23 inhibitors, intra-class switch

## Abstract

**Background:**

Risankizumab is an interleukin (IL)-23 inhibitor that is effective for the treatment of moderate-to-severe Crohn’s disease (CD). However, many patients experience loss of response. Switching within the IL-23 inhibitor class to agents with more frequent dosing, such as guselkumab, may represent a salvage strategy, though no supporting data exist. We aimed to describe outcomes following switching between IL-23 antagonists.

**Methods:**

This case series included five CD patients at a Canadian tertiary care center with primary non-response or secondary loss of response to risankizumab who were switched to guselkumab. Data were collected at baseline, during risankizumab therapy, and after switching.

**Results:**

Five CD patients with inadequate response to risankizumab were included. One patient (20%) had primary non-response and four (80%) had secondary loss of response. Risankizumab duration ranged from 3 to 38 months (mean 17.4 ± 12.9). Four patients had active disease on baseline endoscopy before switching. Mean follow-up after switching was 3.8 ± 1.9 months. Mean CD-PRO2 improved from 22.7 to 5.2 post-switching. Clinical response (≥50% CD-PRO2 reduction) was achieved in 4/5 patients (80%), and clinical remission (CD-PRO2 < 8) was similarly achieved in 4/5 patients (80%). Among four patients with biochemical data, biochemical response (≥50% reduction in fecal calprotectin [FCal]) and remission (normalization of FCal) each occurred in 75%, with a mean FCal reduction of 618 µg/g. One patient had biochemical worsening despite clinical improvement. No safety concerns were reported.

**Conclusion:**

In CD patients with loss of response to risankizumab, switching to guselkumab may be effective in selected patients. Larger confirmatory studies are needed.

## 1. Introduction

Interleukin (IL)-23 inhibitors are effective therapies for inducing and maintaining remission in patients with moderate to severe Crohn’s disease (CD).^1–4^ However, primary non-response and secondary loss of response are common in clinical practice^1^^,^^2^^,^^4^^,^^5^. In the early era of tumor necrosis factor (TNF) antagonists in inflammatory bowel disease (IBD), switching to another TNF antagonist was frequent and had measurable clinical benefit.^6^ Intra-class switching between IL-23 antagonists represents a potential salvage strategy for recapturing response. However, there are no data on this approach. In this case series, we aimed to report outcomes of patients with inadequate response to risankizumab who were subsequently switched to guselkumab.

## 2. Case series

All patients who received risankizumab for the treatment of IBD at our institution were screened for subsequent switch to guselkumab during follow-up through May 2026. Among 176 identified patients, five were switched to guselkumab, all of whom were CD patients. The cohort included two male and three female patients. Mean disease duration was 13.6 years (±12.3 years). Two patients had previously failed TNF antagonists, one patient had experienced failure to a TNF antagonist and ustekinumab, and two patients had no previous advanced therapy. All five patients showed active disease on baseline endoscopy with a mean Simple Endoscopic Score for Crohn’s Disease (SES-CD) score of 15.8 (±6.8 SD). Baseline characteristics of patients are summarized in Table 1.

**Table 1.** Baseline characteristics of patients prior to risankizumab initiation.

Patient	#1	#2	#3	#4	#5
Sex	Male	Female	Female	Male	Female
Age at CD diagnosis (years)	53	28	40	22	20
Disease duration (years)^a^	16	33	0	3	16
Disease location	Colonic	Ileocolonic	Colonic	Colonic	Ileocolonic
Disease behavior	Penetrating	Stricturing and penetrating	Non-stricturing, non-penetrating		
Perianal disease	Yes	No	No	No	No
Prior abdominal surgery	No	Yes	No	No	No
Prior biologic use	Anti-TNF + Stelara	0	0	Anti-TNF	Anti-TNF
Smoking status	Past smoker	Non-smoker	Non-smoker	Non-smoker	Non-smoker
Baseline CRP (mg/L)	14	6.34	31	N/A	3.84
Baseline FCal (µg/g)	96	95	525	N/A	<17
Albumin (g/L)	41	38	24	N/A	N/A
Steroid use at baseline	No	No	No	Yes	No
Baseline endoscopy	Active disease with ulcers	Active disease with ulcers and frank stenosis	Active disease with ulcers	Active disease with ulcers	Active disease with ulcers
Baseline SES-CD	N/A	7	26	15	15

a Disease duration: number of years between diagnosis and IL-23 initiation.

Abbreviation: N/A, data not available; CD, Crohn’s disease; CRP, C-reactive protein; FCal, fecal calprotectin; SES-CD, Simple Endoscopic Score for Crohn’s Disease.

Four patients (80%) experienced secondary loss of response while receiving risankizumab maintenance therapy (360 mg subcutaneously [SC] every 8 weeks), and one patient (20%) demonstrated primary non-response following three induction doses of risankizumab 600 mg intravenously (IV). Two patients (patients #1 and #3) received an additional 600 mg IV dose of risankizumab (see Table 2). Patient #1 received a rescue dose during maintenance therapy for secondary loss of response, with a persistent rectal ulcer identified on follow-up endoscopy. Patient #3 received an additional dose during induction therapy due to persistently elevated C-reactive protein (CRP) after the three scheduled induction infusions.

**Table 2.** Clinical, biochemical and endoscopic outcomes before and after switch from risankizumab to guselkumab.

Patient	#1		#2		#3		#4		#5	
**Duration on risankizumab before switch to guselkumab (months)**	17		3		12		38		17	
**Reason for switch**	Secondary non-response		Primary non-response		Secondary non-response		Secondary non-response		Secondary non-response	
**Duration on guselkumab (months)**	6		3		5		1		4	
**Moment relative to switch**	Before	After	Before	After	Before	After	Before	After	Before	After
**Treatment plan**	Ris: 600 mg IV q4wks × 3 → 360 mg SC q8wks**^a^**	Gus: 200 mg IV q4wks × 3 → 200 mg SC q4wks	Ris: 600 mg IV q4wks × 3 → maintenance not reached	Gus 400 mg SC q4 wks × 3 → 200 mg SC q4wks	Ris: 600 mg IV q4wks × 4**^b^**→ 360 mg SC q8wks	Gus 400 mg SC q4wks × 3 → 200 mg SC q4wks	Ris: 600 mg IV q4wks × 3 → 360 mg SC q8wks	Gus 400 mg SC q4wks × 3 → 200 mg SC q4wks	Ris: 600 mg IV q4wks × 3 → 360 mg SC q8wks	Gus 400 mg SC q4wks × 3 → 200 mg SC q4wks
**Treatment phase**	Maintenance	Maintenance	Induction	Maintenance	Maintenance	Maintenance	Maintenance	Induction	Maintenance	Maintenance
**Abdominal pain (1-10)**	0	0	5	0	0	0	4	0	4-5	0
**Stool frequency per day (consistency)**	3-4 (liquid)	3-4 (formed)	7-8 (liquid)	4-5 (liquid)	4-5 (liquid)	3 (liquid)	4-6 (liquid)	1 (formed)	6-7 (liquid)	1-2 (formed)
**Bloody stool**	No	No	No	No	No	No	No	No	Yes	No
**CD-PRO2 score**	7	7	40	9	9	6	30	2	35.5	3
**Clinical response** ^c^		N/A		Yes		Yes		Yes		Yes
**Clinical remission** ^d^		Yes		No		Yes		Yes		Yes
**CRP (mg/L)**	5.3	5.6	5	8.53	14	10.1	N/A	N/A	N/A	16
**Fecal calprotectin (µg/g)**	1835	215	1671	268	104	699	N/A	N/A	90	46
**Biochemical response** ^e^		Yes		Yes		No		N/A		Yes
**Biochemical remission** ^f^		Yes		No		No		N/A		Yes
**SES-CD**	N/A	N/A	N/A	N/A	19	N/A	7	N/A	N/A	N/A
**Endoscopic appearance**	Proctitis	N/A	Endoscopic recurrence (Rutgeerts i2b)	N/A	Active disease, ulcers	N/A	Active disease with ulcers	N/A	N/A	N/A
**Endoscopic response**		N/A		N/A		N/A		N/A		N/A
**Endoscopic remission**	No	N/A	No	N/A	No	N/A	No	N/A	N/A	N/A
**Histological appearance**	Severe chronic active proctitis	N/A	N/A	N/A	Severe chronic active colitis with erosions and ulcers	N/A	Inflammatory polyps, no significant histologic changes	N/A	Mild chronic active colitis	N/A

a Patient #1 received an additional IV rescue dose during maintenance.

b Patient #3 received an additional IV dose as an extended induction regimen.

c Defined as post-switch reduction in CD-PRO2 score of >50%.

d Defined as post-switch CD-PRO2 score <8.

e Defined as post-switch biomarker (CRP or Fcal) improvement of >50%.

f Defined as post-switch normalization of biomarkers (CRP < 5 mg/L or Fcal < 250 µg/g).

Abbreviations: Ris, risankizumab; Gus, guselkumab; q, every; wks, weeks; CRP, C-reactive protein; SES-CD, Simple Endoscopic Score for Crohn’s Disease.

**Table 3.** Mean clinical and biochemical outcomes before and after the switch to guselkumab.

Outcome	Before	After
Mean CD-PRO2	22.7 ± 15.2	5.2 ± 2.9
Clinical remission (%)	1/5 (20)	4/5 (80)
Mean FCal (µg/g)	925 ± 830	307 ± 241
Biomarker remission (%)	0/4 (0)	3/4 (75)

Abbreviations: CD-PRO2, Crohn’s Disease Patient-Reported Outcome-2; FCal, fecal calprotectin.

The indications for switching to guselkumab varied across patients. Patient #1 had persistent clinical symptoms, elevated fecal calprotectin (FCal), and an endoscopically and histologically active disease on maintenance doses of risankizumab. Patient #2 experienced primary non-response to risankizumab induction, with ongoing symptoms and endoscopic evidence of ulceration, fistulization, and impassable stenosis. Patient #3 was switched due to active disease on endoscopy (SES-CD = 19), with histology confirming severe active chronic colitis with erosions and ulceration. Patients #4 and #5 experienced clinical relapse with recurrence of symptoms at weeks 6-7 on risankizumab maintenance therapy, which prompted the switch to guselkumab with more frequent dosing. Four patients underwent guselkumab induction with three doses of 400 mg SC at 4-week intervals, and one patient received induction with three doses of 200 mg IV. All five patients were subsequently prescribed a maintenance therapy of 200 mg SC every 4 weeks.

### 2.1. Clinical response and remission post switch to guselkumab

Following the switch to guselkumab, all five patients (100%) reported overall clinical improvement within a mean follow-up duration of 3.8 ± 1.9 months. Mean baseline Crohn’s Disease Patient-Reported Outcome-2 (CD-PRO2) score was 22.7 ± 15.2, improving to 5.2 ± 2.9 at follow-up (see Tables 2 and 3).

Clinical response, defined as ≥50% reduction in CD-PRO2 score, was achieved in four out of five patients (80%), and clinical remission (CD-PRO2 < 8) was similarly achieved in four out of five patients (80%). Patient #1 was already in clinical remission prior to the switch and maintained remission throughout follow-up, with subjective improvement in stool consistency despite stable CD-PRO2 scores.

### 2.2. Biomarker response and remission post switch

Biomarker response, defined as a ≥50% reduction in inflammatory biomarker (FCal), was observed in three out of four evaluable patients (75%), while biomarker remission with normalization of FCal (<250 µg/g) was achieved in three out of four patients (75%). Among the four patients with available biochemical data, mean FCal improved from 925 ± 830 µg/g before the switch from risankizumab to guselkumab to 307 ± 241 µg/g after the switch. Mean reduction in FCal was 618 ± 611 µg/g. One patient (#3) did not achieve biomarker response, with FCal increasing from 104 µg/g prior to switch to 699 µg/g after 1 month of guselkumab therapy, although this patient still achieved clinical response and remission.

No post-switch endoscopic data were available at the time of analysis. No adverse events or safety concerns were reported following the switch to guselkumab.

## 3. Discussion

In this case series of five CD patients with loss of response to risankizumab, switching to another IL-23 antagonist, guselkumab, led to improved outcomes. These findings suggest that switching within the IL-23 inhibitor class may be an effective strategy to improve outcomes and achieve remission in selected patients. The proposed rationale for the demonstrated improvement in outcomes is the more frequent dose administration of guselkumab in its maintenance treatment regimen (every 4 weeks) and potential molecular differences.

This is the first limited evidence for switching between IL-23 antagonists in patients with CD, and it is consistent with reported data on intra-class anti-TNF switching in CD as well as literature on intra-class IL-23 inhibitor switching in rheumatological diseases.^7^^,^^8^ Indeed, a meta-analysis on the efficacy of subsequent anti-TNF therapy after initial failure on a different anti-TNF in IBD patients reported an overall clinical remission rate of 43%, with stratification of remission rates based on the reason for the anti-TNF switch demonstrating a stronger response among patients switched due to intolerance (61%) compared to secondary loss of response (43%) and primary non-response (30%).^7^ Most studies included in this meta-analysis reported switching from infliximab to adalimumab. Moreover, our findings are consistent with data reported in a systematic review on the safety and efficacy of intra-class IL-23 inhibitor switching in the treatment of psoriasis,^8^ reflecting the potential use of this approach in patients with CD.

Limitations of this study include its design as a retrospective observational case series and the small cohort size. Moreover, the lack of endoscopic data following the switch to guselkumab limits our capacity to confirm endoscopic response, although demonstrated clinical and biochemical improvement would suggest a correlated response upon mucosal assessment. Nonetheless, this is, to our knowledge, the first study reporting on outcomes in CD patients having undergone intra-class IL-23 inhibitor switching. The improvement of patient outcomes following the switch to guselkumab after primary non-response or secondary loss of response to risankizumab may signal potential efficacy of intra-class IL-23 inhibitor switching, which warrants further validation through high-quality trials. Larger prospective experimental studies with robust clinical, biochemical, and endoscopic data are needed to confirm this signal, to define optimal intra-class switching protocols, and to identify predictors of response.

## Data Availability

The data underlying this article cannot be shared publicly due to patient privacy and institutional ethical restrictions related to retrospective electronic medical record data. De-identified data will be shared on reasonable request to the corresponding author.
